# Analysis of the current status and influencing factors of latent tuberculosis infection among new students in Taishan District in 2025

**DOI:** 10.3389/fpubh.2026.1836962

**Published:** 2026-05-18

**Authors:** Huanqing Bian, Jun Zhou, Xingang Li, Xiuli Yan, Yonghe Sun, Xuebo Liu, Yan Ren

**Affiliations:** Taishan District Center for Disease Control and Prevention, Tai’an, China

**Keywords:** strong positivity rate, influencing factors, latent tuberculosis infection, new students, tuberculosis

## Abstract

**Objective:**

To analyze the current status and influencing factors of latent tuberculosis infection among new students in Taishan District in 2025, providing data support for the formulation of tuberculosis prevention and control strategies in schools.

**Methods:**

A total of 38,863 new students in 2025 were selected as the study subjects and underwent standardized PPD skin tests. Statistical analysis was performed using SPSS software, with group comparisons conducted via chi-square tests, and a multivariate logistic regression model employed to analyze influencing factors.

**Results:**

In 2025, 38,863 new students in Taishan District underwent valid screening, with an overall screening rate of 92.70%. PPD test results showed that 30,438 students (78.32%) were negative, 2,243 (5.77%) were strongly positive, and the total number of latent tuberculosis infection cases was 5,395, with a latent infection rate of 13.88%. Multivariate analysis revealed that male gender (OR = 0.880, 95%CI: 0.829–0.935), junior high school (OR = 0.331, 95%CI: 0.230–0.477), senior high school (OR = 0.471, 95%CI: 0.404–0.550),vocational school (OR = 0.531, 95%CI: 0.457–0.618); junior college (OR = 0.690, 95%CI: 0.644–0.739); new students aged 17–18 years (OR = 0.787, 95%CI: 0.737–0.841), and students with no history of tuberculosis exposure (OR = 0.096, 95%CI: 0.079–0.116) had a relatively lower risk of latent infection.

**Conclusion:**

The prevalence of latent tuberculosis infection and the rate of strongly positive results among new students in Taishan District in 2025 were relatively high. Female students, undergraduate students, those aged 19 years and older, and students with a history of tuberculosis exposure were identified as high-risk groups for latent infection. Screening of incoming new students should be strengthened, and individuals with latent infection should undergo dynamic monitoring and preventive interventions to reduce the risk of tuberculosis occurrence on campus.

## 1 Introduction

Latent tuberculosis infection refers to a condition in which a person infected with *Mycobacterium tuberculosis* does not exhibit clinical symptoms of active tuberculosis or radiological abnormalities. Although individuals with latent infection are not contagious, they remain at risk of developing the disease (1). Studies indicate that approximately one-quarter of the global population is in a state of latent tuberculosis infection, and the risk of latent tuberculosis infection progressing to active tuberculosis is 5–10%. Once latent infection develops into active tuberculosis, it is easily overlooked or misdiagnosed due to the lack of obvious early symptoms. Patients can easily transmit the bacteria to close contacts through coughing, sneezing, and prolonged direct contact. This not only poses a serious threat to human health but also, given the long treatment duration and high costs associated with tuberculosis, places a heavy economic burden on families and consumes significant public health resources, resulting in a substantial disease burden on society as a whole (1, 2). As schools are densely populated settings, the enrollment of a patient with active tuberculosis can easily trigger a cluster outbreak on campus, severely disrupting teaching order and having a significant impact on students’ physical and mental health. Through school entry screening, potential sources of infection and latent tuberculosis carriers can be proactively identified and isolated for treatment, thereby preventing outbreaks and safeguarding the health of teachers and students (3). The prevention and control of latent tuberculosis infection are crucial measures for ending the tuberculosis epidemic. Therefore, this study aims to analyze the 2025 enrollment screening data of new students in Taishan District to understand the current status of latent tuberculosis infection in schools and its associated risk factors, thereby providing a theoretical basis and practical reference for future prevention and control efforts and for effectively reducing the incidence of tuberculosis.

## 2 Subjects and methods

### 2.1 Study population

In accordance with the Chinese School Tuberculosis Prevention and Control Guidelines (2020 Edition) (4), tuberculosis screening was conducted for all incoming students at boarding junior high schools, all senior high schools, and colleges and vocational schools within Taishan District. Schools distributed the “Notice on Tuberculosis Screening (PPD Test) for New Students,” signed and confirmed by parents or the students themselves. A total of 20 institutions participated in the screening of new students, including 5 junior high schools, 6 senior high schools, 2 vocational schools, 4 junior colleges, and 3 universities.

### 2.2 Relevant definitions

Tuberculosis Screening: Conducted by professionally trained personnel, the procedure involves an intradermal injection of 5 IU of PPD reagent (Beijing Xiangrui Biological Products Co., Ltd.) into the anterior third of the palmar side of the subject’s left forearm. The injection site is observed 48–72 h after administration. A result is considered negative if the average diameter of the erythema or induration is <5 mm or if there is no reaction; an induration diameter ≥5 mm is considered positive; specifically, 5 mm ≤ induration diameter < 10 mm is classified as mild positivity; 10 mm ≤ induration diameter < 15 mm is classified as moderate positivity; and an induration diameter ≥15 mm, or the presence of a double ring, blistering, necrosis, or lymphangitis at the injection site, is classified as strong positivity (5, 6). All positive cases underwent further chest X-ray examinations, and no cases of active pulmonary tuberculosis were detected.

Latent Tuberculosis Infection (LTBI): In this study, individuals with an average induration diameter of ≥10 mm on the PPD skin test and no radiographic abnormalities were defined as having latent tuberculosis infection (7). Accordingly, the LTBI group includes individuals with moderate and strong positive reactions.

### 2.3 Quality control

Uniform training was provided to personnel involved in PPD injection and result interpretation to ensure consistency in injection procedures and accuracy in result interpretation. Data were uniformly collected, reviewed, and entered by qualified professionals.

### 2.4 Statistical analysis

Statistical analysis was performed using SPSS 22 software. Differences between groups were assessed using the chi-square tests, and influencing factors were analyzed using multivariate logistic regression. Tests were conducted at the two-sided level with *α* = 0.05.

## 3 Results

### 3.1 Overview of new students screening in Taishan District in 2025

In 2025, the total number of registered new students in Taishan District was 41,922, with 38,863 actually completing the screening, resulting in an overall effective screening rate of 92.70%. A total of 3,059 students did not participate in the screening; the main reasons included screening contraindications, absence on the first day of school, and explicit personal refusal. Among the screened population, there were 18,750 males (48.25%) and 20,113 females (51.75%), with a sex ratio of 1:1.08. In terms of educational level, undergraduate freshmen accounted for the highest proportion (38.00%), followed by junior college students (35.96%), senior high schools students (14.30%), vocational school students (10.48%), and junior high school students (1.25%). The vast majority of students attended public schools (77.80%) and were of Han ethnicity (99.59%). The age group with the largest number of participants was 19 years and older (38.18%). Additionally, 451 individuals (1.16%) had a history of exposure to tuberculosis (Table 1).

**Table 1:** Baseline characteristics of new students screened in Taishan District, 2025.

Category		Number of registered new students (%)	Actual number screened (%)
Gender	Male	20,164 (48.10%)	18,750 (48.25%)
	Female	21,758 (51.90%)	20,113 (51.75%)
Educational Level	Junior high school	557 (1.33%)	487 (1.25%)
	Senior high schools	6,251 (14.91%)	5,559 (14.30%)
	Vocational school	4,528 (10.80%)	4,072 (10.48%)
	Junior colleges	15,145 (36.13%)	13,976 (35.96%)
	Undergraduate	15,441 (36.83%)	14,769 (38.00%)
School Type	Public	32,538 (77.62%)	30,236 (77.80%)
	Private	9,384 (22.38%)	8,627 (22.20%)
Ethnic	Han	41,758 (99.61%)	38,705 (99.59%)
	Ethnic Minorities	164 (0.39%)	158 (0.41%)
Age	16 years old and under	10,592 (25.27%)	9,352 (24.06%)
	17–18 years	15,536 (37.06%)	14,673 (37.76%)
	19 years and older	15,794 (37.67%)	14,838 (38.18%)
History of tuberculosis exposure	Yes	467 (1.11%)	451 (1.16%)
	No	41,455 (98.89%)	38,412 (98.84%)

### 3.2 2025 Taishan District new students screening results

**Figure 1:**
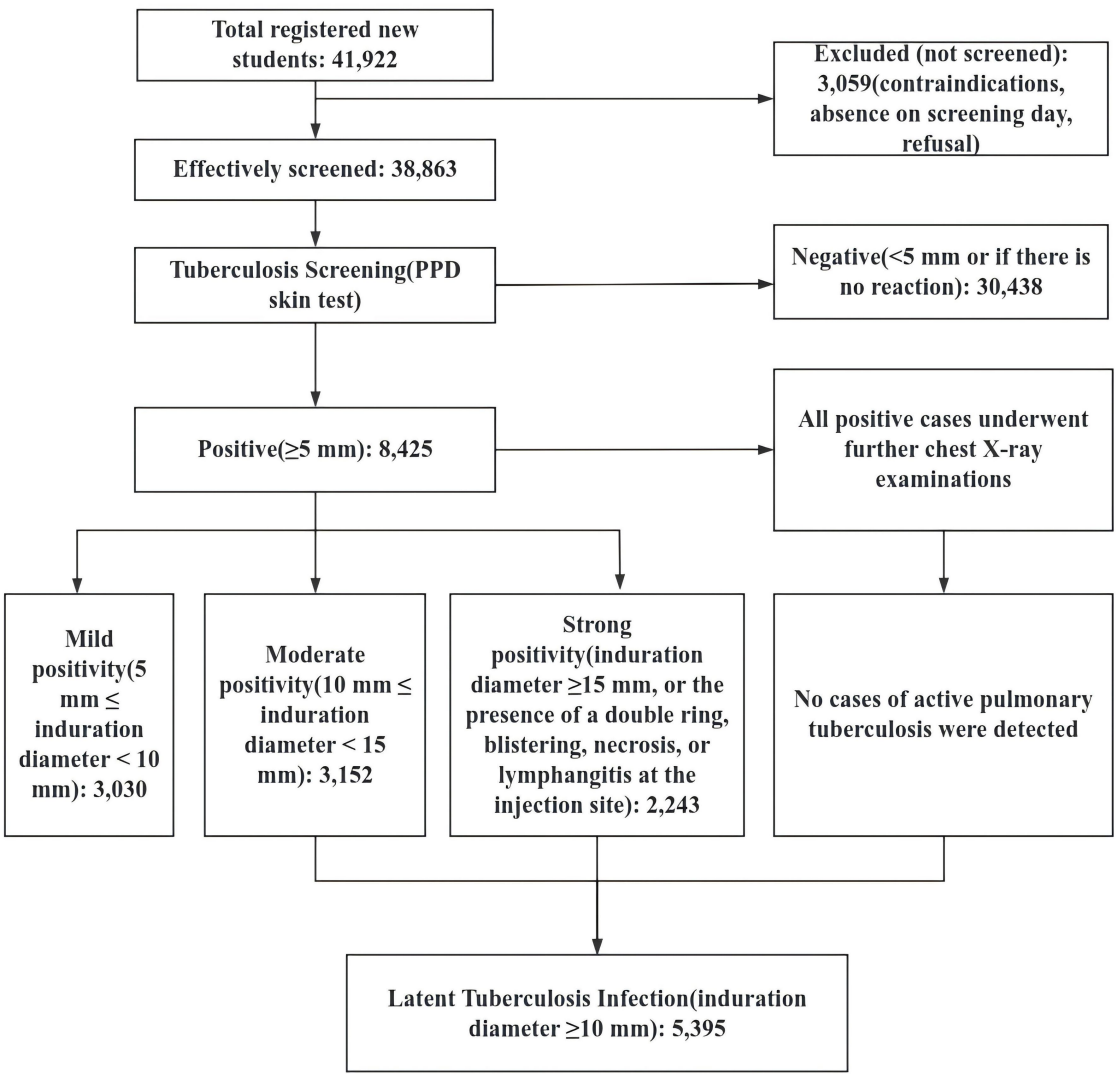
Flowchart of participant screening and inclusion.

Among the 38,863 valid screening subjects, 30,438 had negative PPD reactions, accounting for 78.32%; 8,425 had positive PPD reactions (induration ≥5 mm), with an overall positivity rate of 21.68%. All positive cases underwent further chest X-ray examinations, and no cases of active pulmonary tuberculosis were detected. Among them, 2,243 were strongly positive, with a strong positivity rate of 5.77%. According to the definitions used in this study, there were a total of 5,395 latent infections, with an overall latent infection rate of 13.88% (Figure 1). There were 2,361 male latent tuberculosis cases (prevalence rate: 12.59%) and 3,034 female latent tuberculosis cases (prevalence rate: 15.08%); there was a significant difference in prevalence rates between genders (*χ*^2^ = 50.438, *P* < 0.001). Undergraduate students had the highest latent infection rate at 17.95%, followed by junior college students (12.86%), vocational school students (10.07%), senior high school students (8.92%), and junior high school students (8.21%); there were significant differences in latent infection rates across educational levels (*χ*^2^ = 393.500, *P* < 0.001). The latent infection rate in private schools (15.44%) was higher than that in public schools (13.44%), indicating a significant difference between the two (*χ*^2^ = 22.508, *P* < 0.001). The latent infection rate among Han students was 13.86%, while that among ethnic minority students was 18.35%; the difference in latent infection rates between the two groups was not statistically significant (*χ*^2^ = 2.654, *P* = 0.103). The latent infection rate among students aged 19 and older was 17.15%, among those aged 17–18 it was 13.15%, and among those aged 16 and younger it was 9.85%; the differences in latent infection rates across these age groups were statistically significant (*χ*^2^ = 266.636, *P* < 0.001). There was a statistically significant difference in the latent infection rate among students with and without a history of tuberculosis exposure (*χ*^2^ = 830.619, *P* < 0.001) (Table 2).

**Table 2:** Latent tuberculosis infection status among new students in Taishan District in 2025.

Variable		Number of latent infections	Latent infection rate	*χ^2^*	*P*
Gender	Male	2,361	12.59%	50.438	*P* < 0.001
	Female	3,034	15.08%		
Educational level	Junior high school	40	8.21%	393.500	*P* < 0.001
	Senior high schools	496	8.92%		
	Vocational school	410	10.07%		
	Junior colleges	1,798	12.86%		
	Undergraduate	2,651	17.95%		
School Type	Public	4,063	13.44%	22.508	*P* < 0.001
	Private	1,332	15.44%		
Ethnic	Han	5,366	13.86%	2.654	0.103
	Ethnic Minorities	29	18.35%		
Age	16 years old and under	921	9.85%	266.636	*P* < 0.001
	17–18 years	1,929	13.15%		
	19 years and older	2,545	17.15%		
History of tuberculosis exposure	Yes	273	60.53%	830.619	P < 0.001
	No	5,122	13.33%		

### 3.3 Multivariate analysis of factors influencing latent tuberculosis infection among new students

Univariate analysis revealed that gender, educational level, school type, age group, and history of tuberculosis exposure were factors influencing the prevalence of latent tuberculosis infection among new students during screening. Variables with *P* < 0.05 were included in the multivariate analysis. The results showed that, compared to females, males had a 0.880-fold lower risk of latent tuberculosis infection (OR = 0.880, 95%CI: 0.829–0.935). Compared to the undergraduate level, the risk of latent tuberculosis infection at the junior high school level was 0.331 times that of the undergraduate level (OR = 0.331, 95%CI: 0.230–0.477); the risk at the senior high school level was 0.471 times that of the undergraduate level (OR = 0.471, 95%CI: 0.404–0.550); the risk during vocational school was 0.531 times that of undergraduate studies (OR = 0.531, 95%CI: 0.457–0.618); and the risk during junior college was 0.690 times that of undergraduate studies (OR = 0.690, 95%CI: 0.644–0.739). Students aged 17–18 years had a 0.787-fold lower risk of latent tuberculosis infection compared to students aged 19 and older (OR = 0.787, 95%CI: 0.737–0.841). Compared with students with a history of tuberculosis exposure, those without such a history had a lower risk of latent tuberculosis infection (OR = 0.096, 95%CI: 0.079–0.116) (Tables 3, 4).

**Table 3:** Variable coding for multivariate logistic regression analysis of factors influencing latent infection among new students.

Variable	Categorization
Gender	Male = 0; Female = 1
Education Level	Junior high school = 0; Senior high schools = 1; Vocational school = 2; Junior colleges = 3; Undergraduate = 4
School Type	Public = 0; Private = 1
Age	16 years old and under = 0; 17–18 years old = 1; 19 years old and older = 2
History of Tuberculosis Exposure	No = 0; Yes = 1
Latent Infection	No = 0; Yes = 1

**Table 4:** Multivariate logistic regression analysis of factors influencing latent infection in new students.

Variable		Reference	*β*	SE	Wald *χ*^2^	*P*	OR	95% confidence interval
Gender	Male	Female	−0.127	0.031	17.464	<0.001	0.880	(0.829–0.935)
Educational level	Junior high school	Undergraduate	−1.106	0.187	35.131	<0.001	0.331	(0.230–0.477)
	Senior high schools		−0.752	0.079	90.229	<0.001	0.471	(0.404–0.550)
	Vocational school		−0.632	0.077	66.817	<0.001	0.531	(0.457–0.618)
	Junior colleges		−0.372	0.035	111.464	<0.001	0.690	(0.644–0.739)
School Type	Public	Private	−0.053	0.038	1.941	0.164	0.948	(0.880–1.022)
Age	16 years and under	19 years and older	−0.101	0.069	2.144	0.143	0.904	(0.789–1.035)
	17–18 years		−0.127	0.034	50.122	<0.001	0.787	(0.737–0.841)
History of tuberculosis exposure	No	Yes	−2.345	0.099	556.105	<0.001	0.096	(0.079–0.116)

## 4 Discussion

As a global public health issue, the prevention of latent tuberculosis infection is a crucial measure for ending the tuberculosis epidemic. Approximately one-quarter of the global population are latent tuberculosis carriers; without intervention, a portion of these individuals will develop active tuberculosis. Therefore, screening for latent infection and providing preventive treatment not only shifts the focus of control efforts to an earlier stage but also eliminates the epidemic at its source, laying the foundation for the goal of ending the tuberculosis epidemic by 2035 (8, 9). Schools, with their high population density, are high-risk areas for cluster outbreaks (10). As a key target group for tuberculosis control, students should undergo enhanced active screening to detect latent infections early and effectively interrupt the chain of transmission.

The results of this study show that the rate of strongly positive tuberculosis test results among new students in Taishan District was 5.77%, higher than that of Shanxi Province (0.79%) (11). The rate of latent infection was 13.88%, higher than that of Shanghai (4.8%) (12) and Nanjing (13.63%) (13). Although there are regional variations in the strong-positive rate and latent infection rate among newly enrolled students, both indicators in Taishan District are at relatively high levels, indicating that students in this region face a more severe risk of exposure to *Mycobacterium tuberculosis* and a potential burden of tuberculosis incidence. Possible reasons include the following: First, as the central urban district of Tai’an City, Taishan District is densely populated and serves as an educational hub, attracting students from various regions, which increases the risk of *M. tuberculosis* transmission and cross-infection. Second, some urban schools are outdated, with inadequate ventilation in classrooms and dormitories, which facilitates the spread of the bacteria. Third, cross-reactions resulting from BCG vaccination contribute to a certain extent to the high rate of strong positive results (14, 15).

This study shows that men have a lower risk of latent tuberculosis infection than women, which is consistent with survey findings indicating a higher rate of LTBI among women than men in Changshou District, Chongqing (16), as well as the conclusions of studies by Wada et al. (17). The physiological mechanism underlying this is that female macrophages possess stronger initial phagocytic and uptake capabilities against *M. tuberculosis*, making it easier for latent infection to develop; relevant hormones in the body can inhibit the proliferation of the pathogen, which, while reducing the risk of progression to active tuberculosis, has no significant effect on the occurrence of latent infection (18). Additionally, women are more likely to spend time in crowded settings such as classrooms and dormitories. Prolonged exposure to poorly ventilated, enclosed environments increases the risk of indoor transmission of *M. tuberculosis*, thereby raising the probability of LTBI (19, 20). Furthermore, female freshmen experience higher levels of psychological stress during the initial phase of enrollment; prolonged stress can weaken the body’s immune function, further increasing the potential risk of *M. tuberculosis* infection (21).

The results of this study indicate that LTBI risk is higher among undergraduate freshmen. Undergraduate freshmen come from a wide range of regions, spanning areas with significantly varying levels of tuberculosis prevalence. Their broader social contacts provide conditions for exposure to latent *M. tuberculosis* infection (22). This group is in the transitional phase from adolescence to adulthood. Accompanied by physical development and endocrine fluctuations, coupled with changes in diet and living environments, their immune systems are in a dynamic period of adaptation and adjustment. Consequently, the efficacy of their immune defenses is prone to fluctuations, thereby increasing susceptibility to LTBI (23). Furthermore, having just completed the college entrance exams, these students face significant academic pressure, which can lead to relatively weakened immune function. Additionally, the concentrated and intensive nature of undergraduate coursework and dormitory life creates a relatively closed, densely populated microenvironment that significantly increases the probability of *M. tuberculosis* transmission. The combined effect of these multiple factors makes undergraduate freshmen a high-risk group for LTBI (24, 25).

Age is also a significant factor influencing the rate of latent tuberculosis infection among new students (26). The results of this study indicate that new students aged 19 and older have a higher risk of latent tuberculosis infection than those aged 17–18, which may be related to the cumulative exposure opportunities that increase with age. Older new students, due to their longer life span, have a higher probability of exposure to active pulmonary tuberculosis cases in family, school, and social settings, thereby leading to an increased risk of latent infection (27).

New students with a history of tuberculosis contact have a higher risk of latent infection, which is similar to the findings of Guo et al. (28). This is because new students with a history of tuberculosis exposure—particularly those who have had prolonged, close contact with active pulmonary tuberculosis patients (such as family members) in enclosed spaces-face a significantly increased risk of inhaling *M. tuberculosis* droplets through the respiratory tract, making them highly susceptible to primary infection (29). Therefore, schools should prioritize these students for health monitoring and strengthen screening and follow-up during the enrollment process to effectively prevent latent infections from progressing to active tuberculosis.

## 5 Conclusion

In summary, the burden of latent tuberculosis infection among new students in Taishan District in 2025 is relatively high, and its distribution exhibits a distinct clustering pattern within specific populations. Female students, undergraduate students, students aged 19 years and older, and new students with a history of tuberculosis exposure constitute high-risk groups for latent infection. It is recommended to implement targeted prevention and control measures, including strengthening preventive treatment and follow-up for high-risk groups, enhancing supervision of tuberculosis prevention efforts in undergraduate institutions, and strictly enforcing campus ventilation protocols to reduce the risk of tuberculosis transmission.

## Data Availability

The original contributions presented in the study are included in the article/supplementary material, further inquiries can be directed to the corresponding author.
